# Comparison of Behavior and Space Use of the European Bullhead *Cottus gobio* and the Round Goby *Neogobius melanostomus* in a Simulated Natural Habitat

**DOI:** 10.3390/biology10090821

**Published:** 2021-08-24

**Authors:** Sara Roje, Bořek Drozd, Luise Richter, Jan Kubec, Zdeněk Polívka, Susanne Worischka, Miloš Buřič

**Affiliations:** 1 South Bohemian Research Center of Aquaculture and Biodiversity of Hydrocenoses, Faculty of Fisheries and Protection of Waters, University of South Bohemia in České Budejovice, Zátiší 728/II, 389 25 Vodňany, Czech Republic; drozd@frov.jcu.cz (B.D.); kubecj@frov.jcu.cz (J.K.); polivz00@frov.jcu.cz (Z.P.); buric@frov.jcu.cz (M.B.); 2 Institute of Hydrobiology, Technische Universität Dresden, 01062 Dresden, Germany; Luise.Richter2@tu-dresden.de; 3 Stream Ecology Lab, Institute of Integrated Natural Sciences, University Koblenz-Landau, Universitätsstrasse 1, 56070 Koblenz, Germany; sworischka@uni-koblenz.de

**Keywords:** habitat overlap, cottiid, gobiid, biological invasion, shelter occupancy, water velocity

## Abstract

**Simple Summary:**

Invasive round goby and native European bullhead are bottom-dwelling species that occupy a similar niche and are expected to compete for similar resources. Knowledge of how species behave in novel conditions without competition has implications for how they will interact. Our objective was to investigate the space use, flow velocity preferences and tolerance, shelter use, feeding behavior, and activity patterns of specimens of both species to better understand their basic behavior characteristics. Space in a habitat simulator system was divided for purposes of analysis into seven zones among which fish could move freely. We observed individual fish during acclimatization periods and normal behavior periods of darkness and light. The results showed few differences between species. Round goby showed a preference for areas with slow running water, whereas the bullhead gravitated to higher velocity waters. The species exhibited similar patterns in time spent in zones in a given period, spending most of the time in the shelter and the mid-stream zone. Despite the low differences observed, we can conclude that the fish needed a long acclimatization period for behavior stabilization, which should be considered in future studies.

**Abstract:**

The round goby is an invasive fish in Europe and North America that threatens native species by predation and competition. Its habitat preferences are similar to those of the European bullhead, which it displaces from shelters and out-competes for available resources. We assessed the microhabitat preferences, shelter use, and activity of the round goby and European bullhead in single-species experiments in habitat simulator systems to investigate their behavior in a novel environment. Fish were video-recorded for 28 h in the presence of shelter and feed with water velocity ranging from 0.00 to 0.96 m s^−1^. The two species showed similar behavior under given conditions. A primary difference was in stress-induced behavior in the initial phases of observation. The round goby spent more time in movement when outside the shelter and a longer time in the escape zone in the exploration period during light. Our results confirmed a significant preference of round goby for low velocity areas and a preference for higher velocities in the European bullhead. Both species were able to cope with velocities > 0.7 m s^−1^. Therefore, the reported invasion success of round goby is probably not driven by space use or activity patterns, but rather by higher adaptability.

## 1. Introduction

Freshwater ecosystems increasingly face native species decline and extinctions and biodiversity loss as a consequence of bioinvasions [1,2]. Direct competition between native and non-native species is a main driver of species replacement, with the latter often more active, aggressive, and prolific, and hence more successful in acquiring and holding resources [3,4]. The differences in ecological preferences and tolerances of interacting species can allow abiotic conditions to modify their competition [5]. In freshwater systems, factors such as hydrodynamics, temperature, slope, habitat complexity, and type of substrate may influence the outcome of direct interactions [6,7,8] and affect the displacement of one species by another, their co-existence in a location, or their separation in space [9]. Consequently, it is necessary to examine the potential results of interactions of invasive and native organisms under the varied abiotic conditions, beginning with obtaining information with respect to species basic behavior patterns, space use, and habitat preferences.

Monitoring of the Ponto-Caspian round goby *Neogobius melanostomus* (Pallas 1814) invasion success [10,11,12] suggests problematic contacts with populations of the vulnerable and endangered European bullhead *Cottus gobio* (L.). The round goby has been one of the most successful non-native fish invaders in European and North American inland and coastal waters in recent decades [13,14]. The species has invaded and expanded its range in large European rivers, including the Danube (possibly natural dispersal), Rhine, Vistula, Volga, and Elbe [15,16,17,18,19,20], expanding into their upper stretches and larger tributaries [12,21,22,23].

The round goby is a small bottom-dwelling species usually associated with crevice habitats [24] and is generally territorial and aggressive [25,26]. Competition with native European and North American species of the family Cottiidae (sculpins) of similar size, environmental requirements, and biology is reported, as both species have the same spawning ground preferences, feeding areas and food types [3,27,28].

The European bullhead is protected under the European Habitat Directive (HD; Annex II, 92/43/EEC) and is an important target species for conservation according to the European Water Framework Directive (WFD; 200/60/EC). It is representative of species potentially affected by round goby invasion [29,30,31]. The European bullhead occurs in freshwater streams, rivers, and lakes with hard stony substrate and shows a preference for shallow fast-flowing water bodies [9]. Its populations are reported in large rivers living in syntropy with the round goby [23,32], implying that they share an ecological niche and directly compete for resources and space in co-inhabited ecosystems. Natural or artificial stones such as ceramic tiles in rivers are vital to assure viable sculpin populations [33]. The nocturnal habits of European bullhead are confirmed [34,35] with solitary bullheads spending the majority of time in shelter during daylight hours [28]. The round goby is also considered nocturnal, with less shelter occupancy in periods of darkness [36,37].

Reported declines in the European bullhead populations [38] coinciding with round goby invasions of the Danube [39] and Rhine [40], along with similar reports for related species from North America [4,41], suggest that Ponto-Caspian gobies can have an adverse impact on sculpins. The round goby and European bullhead were recently reported to co-occur in the Elbe River near the border of the Czech Republic and Germany and in the lower stretches of its tributaries [12], and moreover in at least three Saxon tributaries (Worischka personal observation).

How the interaction of these species is impacted by such habitat conditions as water velocity, shelter, availability of food, and space for surviving is unknown. In case of increased habitat complexity higher abundances of both species are expected, but on the other hand high complexity habitat could reduce predation and competition, allowing more shelter space for the species to occupy. Observations of basic behavior patterns under simulated natural conditions in single-species trials may reveal species space use and preferences in a novel environment unrestricted by the presence of a competitor species.

The present study aimed to characterize round goby and European bullhead light/dark behavior with respect to water flow velocity, shelter availability, and feeding. We hypothesized (1) a higher activity level of round goby than of European bullhead during all 28 h duration of the experiment, (2) more rapid adaptation of the round goby to the novel environment, (3) species-specific flow velocity preferences; European bullhead would use faster flowing areas and round goby would use low velocity areas, (4) difference in activity modes of species depending on light conditions, and (5) greater food intake of the round goby.

## 2. Materials and Methods

### 2.1. Animal Acquisition and Maintenance

Round goby specimens were collected using a backpack pulsed-DC electrofishing unit (FEG 1500, EFKO, Leutkirch, Germany) in April 2019 from recently colonized sites in the Elbe River near Dĕčín, Czech Republic. The site was electrofished during the day based on accessibility for a set amount of time (15 min), working upstream and sampling from bank to bank covering all microhabitats (total 100 m) and targeting smaller fish species. Each site was finished once by four people, one using the anode and the others assisting in the collecting the stunned fish with a dip-net and transporting them to a big box in which fish were later transported to the Institute. The collecting site had a rocky bankside, bottom covered by stones, sand and aquatic vegetation. The European bullheads were collected by electrofishing based on a permission of the Regional Authority of the South Bohemia Region (No. OZZL 104213/2018/pedo SO 2) in Vyšší Brod (Vltava River, Czech Republic, GPS coordinates: 48°37′11.4′′ N, 14°18′51.8′′ E). All bullheads originated from stable, long-term existing populations that represent naive populations unaffected by *N. melanostomus* presence. The capture locality represents riffle with water depth 0.1–0.5 m and riverbed formed by a mixture of stones (particle diameter = 0.1–0.5 m) and gravel (particle diameter = 0.03–0.1 m). All bullheads were released at the capture locality after experiments termination.

Fifty round gobies and thirty European bullheads were transferred to the experimental facility of the Research Institute of Fish Culture and Hydrobiology in Vodňany, the University of South Bohemia in České Budějovice, Czech Republic. The species were held separately in two identical recirculation aquaculture systems. Each species was stocked in two rectangular trays each filled with 240 L aged tap water and equipped with an excessive number of shelters (>3 per individual). A retention tank (filled with approx. 1000 L aged tap water) with a filtration unit and pump supplied the water for the trays and enabling constant water flow. Every three days, 1/3 of the water was exchanged for fresh aged tap water and checked for pH (7.8–8.0). Fish were acclimatized to the temperature of 16 °C for 30 days using cooling system (JDK Dixell XR20CX) with a light regime of 12:12 L:D prior to the beginning of the experiment. Acclimation tanks were structured to prevent fish escape and placed indoors with no direct connection to surface waters to prevent unwanted escape of round goby to natural environment. During the acclimation period, fish were fed ad libitum with common flesh-fly *Sarcophaga carinaria* (L.) larvae daily. Uneaten food was removed by siphoning.

### 2.2. Experimental Setup

Thirty trials (n = 15 fish per species) were conducted in May and June 2019. The experimental setup comprised three independent recirculation systems, each consisting of a channel 2000 mm × 310 mm × 390 mm (L × W × H), a 2000 mm × 510 mm × 400 mm (L × W × H) water retention tank containing aged tap water, and a recirculation pump (WILO IPL 80/1, WILO SE, Dortmund, Germany). Adjustable valves and bypasses enabled regulating the flow velocity.

**Figure 1:**
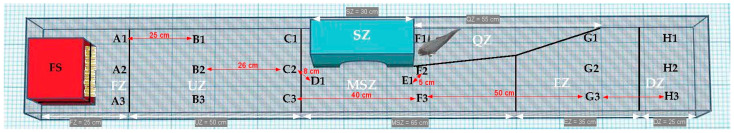
Schematic of the experimental channel showing monitored zones (white letters) FZ = feeding zone where FS = feeding station was situated, UZ = upstream zone, MSZ = mid-stream zone, SZ = shelter zone, QZ = quiet zone, DZ = downstream zone, and EZ = escape zone; points for flow velocity measurement (black numbers A1–H3). Zones are delineated by black lines. Distance between points for flow velocity measurement is noted by red color and distance between monitored zones is marked with white letters on grey background.

Experiments were conducted at 16 °C water temperature. Before each trial, flow velocity was measured at 20 points in the channels by a Flowmeter (MiniController MC20 with the Flowprobe for MiniWater20, Schiltknecht Messtechnik AG, Schaffhausen, Switzerland) to characterize water flow variations throughout the channel and ensure similar conditions in all channels (Figure 1, Table S1).

Each experimental system was equipped with an 8 cm concrete cube located in the upstream area of the system that served to regulate, buffer, and dissipate the strong turbulent flow (water height: 18 cm) at the channel inlet as well as being a feeding station that formed the beginning of the feeding zone (Figure 1). Fifteen *S. carinaria* larvae were attached to fishing line wrapped around the cube, requiring fish to actively pluck the prey from the line. A second concrete block (8 cm × 16 cm), placed approximately in the middle of the tank blocked the flow to provide a quiet zone, and a 10 cm × 8 cm × 4 cm (L × W × H) cavity cut into the lower side of the block furnished shelter. Each experimental channel was divided into: FZ—feeding zone where FS—feeding station was situated, UZ—upstream zone, MSZ—mid-stream zone, SZ—shelter zone, QZ—quiet zone, DZ—downstream zone, and EZ—escape zone (Figure 1).

Fish were approximately measured before each trial and separated for the starving, to have comparable sizes of both species. Specimens with a wet-weight difference of <5% were used in the experiment (Table 1). Fish were unfed for 24 h prior to stocking in the channel. The channels were monitored by a camera system (iGET Homeguard HGDKV-87704, 1080P 3 Channel Digital Video Recorder) attached above experimental channels and connected to a computer. Photoperiod was simulated in order to keep light/dark conditions without the changes during the experimental trials. Permanent indirect illumination was provided by fluorescent tubes (daylight, 2310 lm). Fish were video-recorded for 28 h, from 10.00 h on day one to 14.00 h on day two. We design acclimatization periods (stress response, exploration in light and dark) separated from normal behavior periods in dark and light. Hence, five time periods within the 28 h were analyzed: 1—stress response period (SR), 10:00–14:00 h; 2—exploration period in light (EPL), 14:00–18:00 h; 3—exploration period in dark (EPD), 18:00–22:00 h; 4—normal behavior in dark conditions (NPD) for 8 h, 22:00–06:00 h; and 5—normal behavior in light conditions (NPL) for 8 h (06:00–14:00 h). Each fish was used only once, and after use, all European bullhead specimens were acclimatized to outside ambient temperature for seven days and released into their place of origin.

Fish were weighed using a precision digital balance (Kern 572-35, Kern and Sohn, Germany) to the nearest 0.1 g, and total (TL) and standard length (SL) were measured with a ruler to the nearest 1 mm. Sex of the round gobies was determined based on anal papilla shape. We were unable to determine sex of *Cottus gobio* because of absence of a reliable sex distinguishing method (based on external morphological appearance) without the need of fish sacrifice and gonads inspection. The number of uneaten larvae was counted after each trial, and a new line with fresh larvae was prepared for the subsequent trial.

Video-recordings were analyzed using the automatic ethological software EthoVision^®^ XT software 13.0 (Noldus Information Technology, Wageningen, The Netherlands) that recognizes, tracks, and analyzes the behavior, movement, and activity of fishes. Video-recordings were subsequently checked visually and adjusted/trimmed if errors interfered with detection of fish movement. This is a crucial step at the beginning of data collection, since tracking errors affecting multiple sample points can indicate a problem with the experimental set-up, camera set-up, arena settings, trial control settings, and/or detection settings. The active movement during the trial, distance moved, time spent outside the shelter, time spent in motion outside of the shelter, and time spent in a specific channel zone (Figure 1) were recorded in all time periods.

### 2.3. Statistical Analyses

Data obtained from EthoVision XT 13.0 software were exported to Excel files and analyzed by R software (R Development Core Team, v. 4.0.3., 2020), with the package ggplot2 used for data visualization. Video-tracked behavior patterns included shelter occupancy and space preference, preferred and avoided channel zones, preferred and avoided flow velocities, time spent in motion, distance moved, and number of larvae consumed.

Data were checked for normality and homoscedasticity with Shapiro–Wilks and Bartlett’s tests, respectively. When criteria were met, one-way ANOVA was employed to compare water velocity at each of the 20 points among the three experimental channels. Because data showed non-normal distribution even after transformation, the Mann–Whitney U test (Wilcoxon test) was used to determine differences in the number of larvae eaten by the European bullhead and round goby. The ANCOVA with Tukey’s post hoc test was used to compare the total number of larvae consumed by each species relative to time spent in the feeding zone. A simple linear relationship was used between number of fly larvae consumed and total time spent in feeding zone by round goby and European bullhead. Kruskal–Wallis tests were used to compare the time that an individual fish spent in each zone during a given time period. Wilcoxon test with Bonferroni correction applied to the significance level was used for species comparison of the time spent in each of the seven zones. Mean distance moved (cm), time spent outside the shelter (in seconds and %), time spent in motion outside the shelter (in seconds and %), and total time of active movement during the trial were calculated separately for each species. Results were considered significant at *p* ≤ 0.05.

## 3. Results

### 3.1. Water Velocity

Water velocity in the channel ranged from no velocity 0.00 to 0.96 m s^−1^ (min-max) with no significant difference among channels at any velocity measurement point (ANOVA _(2, 57)_, F = 0.33, *p* = 0.968) (Table S1).

### 3.2. Food Intake

There was no significant difference in the number of larvae consumed with respect to species (Wilcoxin test = 116.5, *p* = 0.8833) (Table 1). A significantly higher number of larvae consumed relative to the time spent in the feeding zone was observed in the round goby compared to the European bullhead (ANOVA _(1, 26)_, F = 4.230, *p* = 0.0499). The time spent in the feeding zone was not significantly related to the number of larvae consumed in either species (European bullhead: Y = 3.47x + 0.00060, R^2^ = 0.1704, *p* = 0.07; round goby: Y = 4.20x + 0.00046, R^2^ = −0.017, *p* = 0.398) (Figure S1). The mean water velocity in the feeding zone was 0.71 m s^−1^, demonstrating that both species successfully coped with high velocities.

### 3.3. Spatial Pattern in Fish Diurnal/Nocturnal Activity

The activity of the European bullhead and the round goby was expressed as duration of movement during the entire 28 h trial, or per hour in cases of different length of time periods in the seven channel zones.

**Figure 2:**
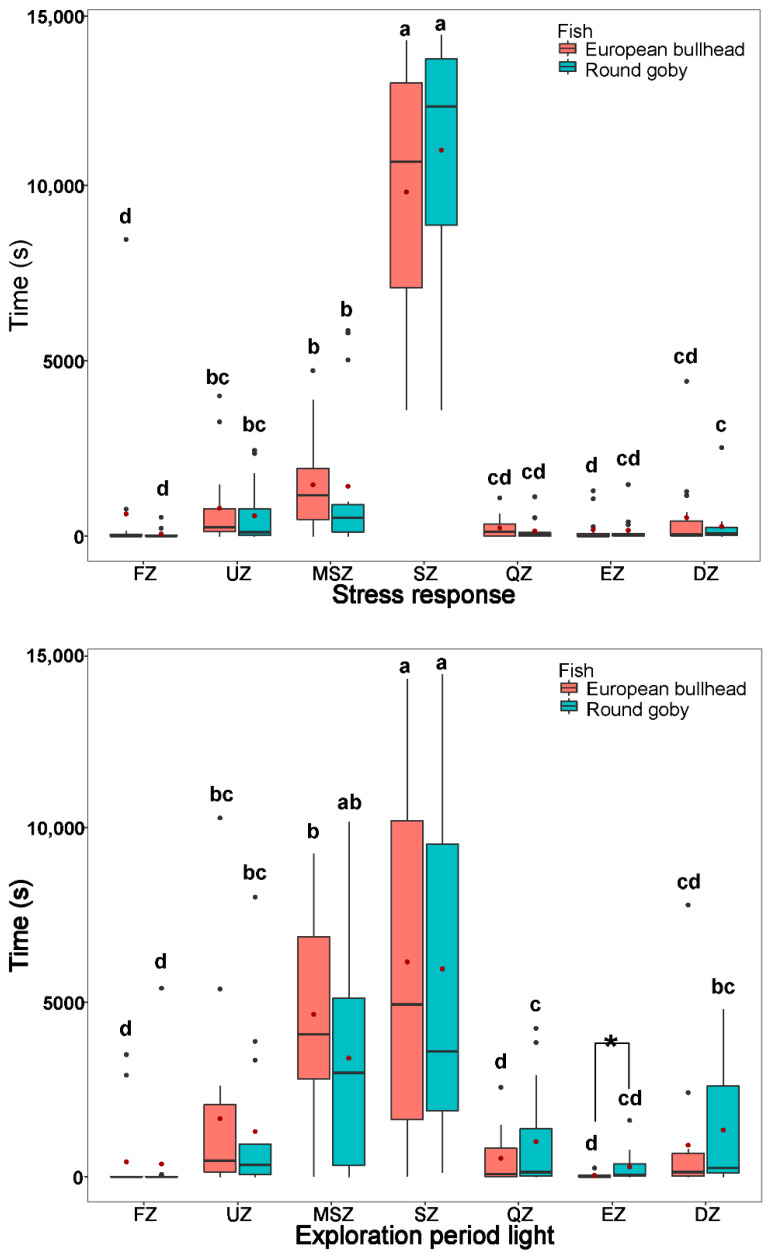

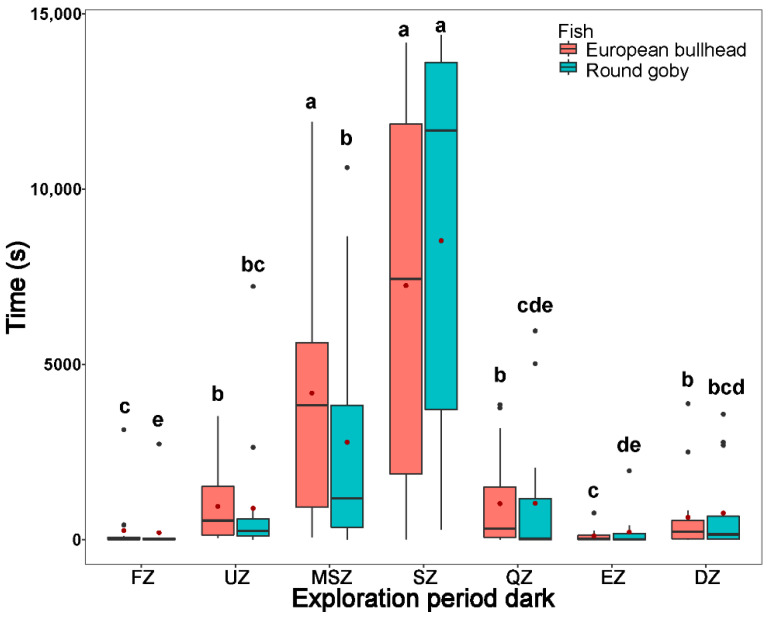
Time spent by *Cottus gobio* (red) and *Neogobius melanostomus* (turquoise) in channel zones during acclimatization periods: stress-response period (upper box plot), exploration period in light (middle box plot), and exploration period in dark (lower box plot). Box limits correspond to upper (Q3—75%) and lower (Q1—25%) quartiles, horizontal bar to the median, and red dot to the mean. Outliers are indicated by points (min = Q1 − 1.5 × IQR; max = Q3 + 1.5 × IQR). FZ = feeding zone, UZ = upstream zone, MSZ = mid-stream zone, SZ = shelter zone, QZ = quiet zone, EZ = escape zone, DZ = downstream zone. Values with different letters indicate significant differences (α = 0.05); asterisk indicates significant interspecific differences.

**Figure 3:**
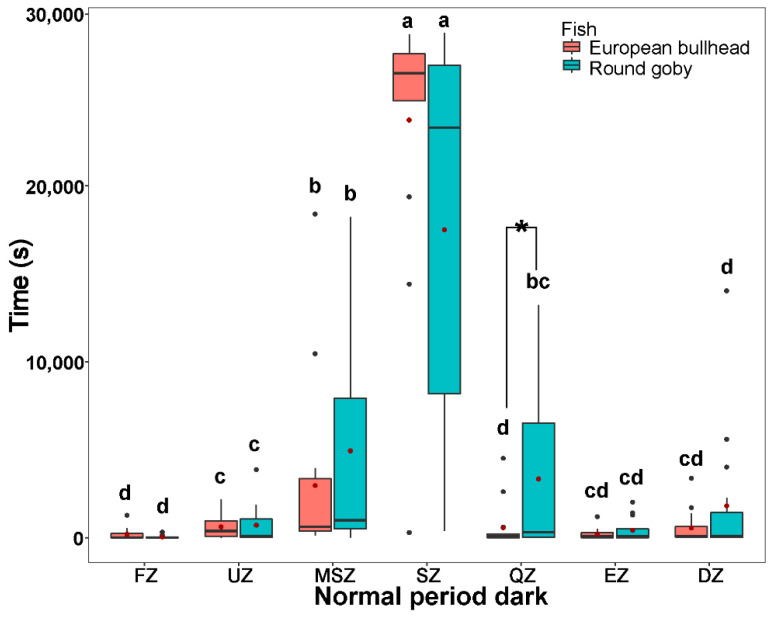

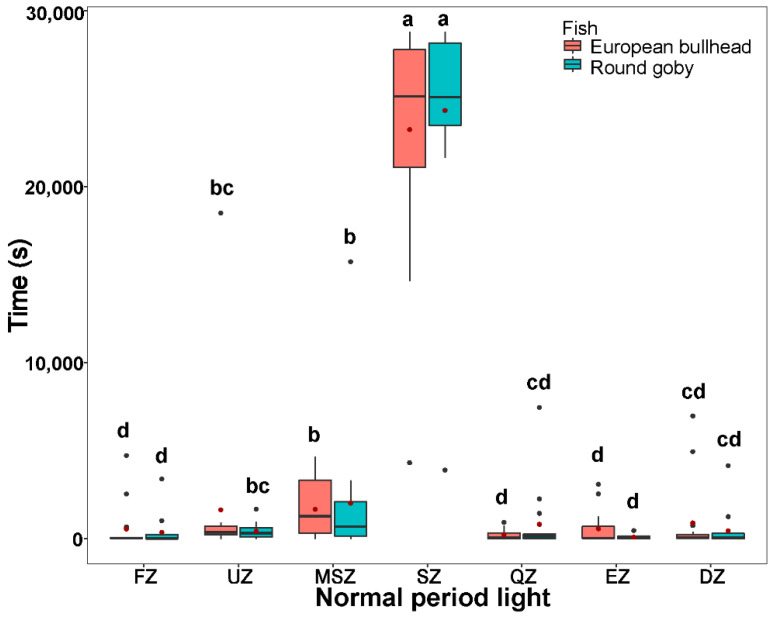
The time spent by *Cottus gobio* (red) and *Neogobius melanostomus* (turquois) in channel zones during two periods of tracking normal behavior in darkness (upper box plot), and light (lower box plot). Box limits correspond to upper (Q3—75%) and lower (Q1—25%) quartiles, horizontal bar to the median, red dot to the mean, outliers are indicated by points (min = Q1 − 1.5 × IQR; max = Q3 + 1.5 × IQR). FZ = feeding zone, UZ = upstream zone, MSZ = mid-stream zone, SZ = shelter zone, QZ = quiet zone, EZ = escape zone, DZ = downstream zone. Values with different letters indicate significant intraspecific differences (α = 0.05); asterisks indicate significant interspecific differences.

The species differed significantly with respect to time in a given zone (Figure 2 and Figure 3). During acclimatization periods (stress response: round goby χ^2^_(6)_ = 51.21, European bullhead χ^2^_(6)_ = 54.071; exploration period light: round goby χ^2^_(6)_ = 35.42, European bullhead χ^2^_(6)_ = 48.32, and exploration period dark: round goby χ^2^_(6)_ = 42.56, European bullhead χ^2^_(6)_ = 47.03 (*p* < 0.001, n = 15)) the species showed a similar pattern of movement, with the greatest difference being in time spent in each zone followed by more or less lively movements (Figure 2). Both species spent most of their time in the shelter and mid-stream zone with average water velocity of ~0.29 m s^−1^, then upstream and downstream zones with water velocity ~0.30 m s^−1^, with the least time spent in the quiet zone with velocity ~0.02 m s^−1^, escape zone at ~0.39 m s^−1^, feeding zone at 0.7 m s^−1^ (Figure 2). The only significant difference between species was during the exploration period light when the round goby was more significantly active in the escape zone (European bullhead 42.4 ± 66.3, round goby 289.5 ± 437.9) (*p* = 0.046) (Figure 2).

During the normal behavior periods in dark and light (normal period dark: round goby χ^2^_(6)_ = 44.66, European bullhead χ^2^_(6)_ = 51.51; normal period light: round goby χ^2^_(6)_ = 47.83, European bullhead χ^2^_(6)_ = 50.41 (*p* < 0.001, n = 15)) patterns of movement in zones were similar to those in the acclimatization periods, with the round goby showing more activity in moving but not statistically significant through the shelter zone during the normal behavior period in darkness than did the European bullhead, which showed more targeted movement in this zone (Figure 3). In the normal behavior period in light, the European bullhead was more active than the round goby, but both species moved less than in other time periods (Figure 3, Table 2). The primary difference between species in normal period dark was significantly more time spent in the quiet zone by the round goby (European bullhead 569.4 ± 1235.6, round goby 3344.9 ± 4644.5) (*p* = 0.033).

During acclimatization periods, the European bullhead moved longer distances than the round goby, which moved greater distances in the normal behavior period in darkness (Table 2). The round goby showed more time in movement during the time periods, with the exception of stress response, than the European bullhead, but differences were not significant (Table 2). During acclimatization, the European bullhead spent more time outside the shelter in the stress response and exploration period in light compared to the round goby, which spent more time outside shelter during the exploration period in dark (Table 2). During the normal behavior periods, the round goby spent more time outside the shelter in light compared to the bullhead, while time spent outside the shelter during darkness was similar in both species (Table 2).

### 3.4. The Temporal Pattern in Fish Diurnal/Nocturnal Activity

There were no significant differences in time feeding (round goby χ^2^_(4)_ = 0.4; European bullhead χ^2^_(4)_ = 5.93) and in upstream (round goby χ^2^_(4)_ = 0.9; European bullhead χ^2^_(4)_ = 1.58), mid-stream (round goby χ^2^_(4)_ = 4.7; European bullhead χ^2^_(4)_ = 9.5), quiet (European bullhead χ^2^_(4)_ = 4.4), downstream (round goby χ^2^_(4)_ = 3.1; European bullhead χ^2^_(4)_ = 1.6), and escape zones (round goby χ^2^_(4)_ = 3.4, n = 15; European bullhead χ^2^_(4)_ = 5.2) between species with respect to time period (*p* > 0.05, n = 15).

**Figure 4:**
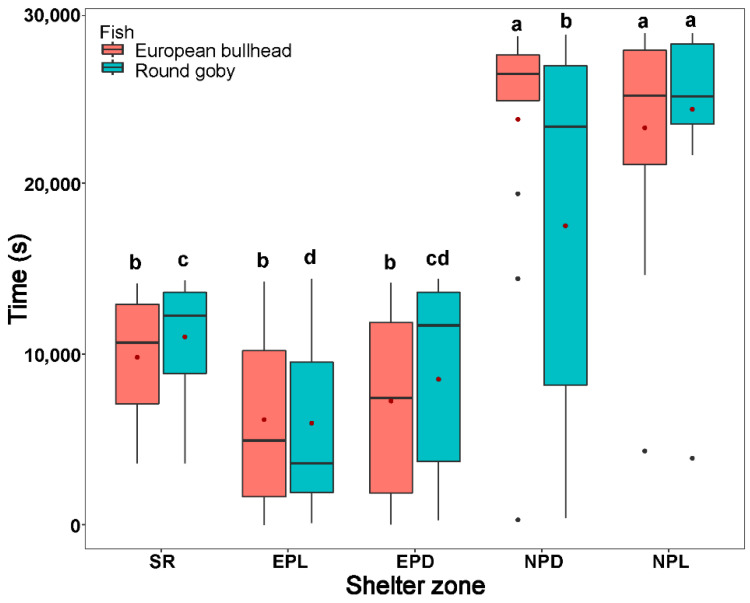

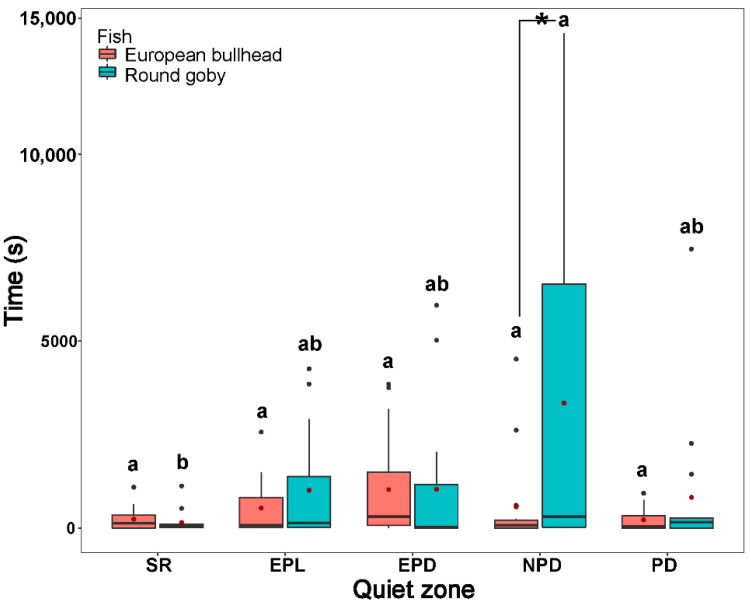
The time in shelter and quiet zones during the stress response period (SR), exploration period in light conditions (EPL), exploration period in dark conditions (EPD), normal behavior period in darkness (NPD), and normal behavior period in light (NPL) by the European bullhead and the round goby in a 28 h trial. Box limits correspond to upper (Q3—75%) and lower (Q1—25%) quartiles, horizontal bar to the median, and red dot to the mean. Outliers are indicated by points (min = Q1 − 1.5 × IQR; max = Q3 + 1.5 × IQR). Different letters indicate significant intraspecific differences (α = 0.05). Asterisks indicate significant interspecific differences.

Both the round goby and European bullhead spent a significantly longer time in shelter during all time periods than in other zones (round goby χ^2^_(4)_ = 23.397; European bullhead χ^2^_(4)_ = 44.148 (*p* < 0.001, n = 15)) (Figure 4). The round goby spent significantly more time in the quiet zone (round goby χ^2^_(4)_ = 5.6, *p* = 0.049, n = 15) than did the European bullhead during the normal period in dark.

## 4. Discussion

This study presents evidence of a similar behavior pattern in a native (European bullhead) and an invasive (round goby) benthic fish species in single-species laboratory experiments. The Ponto-Caspian invader, the round goby, continues to spread in European and North American freshwater and coastal ecosystems and invades vulnerable tributaries of main waterways where it threatens native fish species, competing with them for habitat and prey [3,36,42]. Examples of vulnerable native fish species negatively affected by the round goby are the log-perch *Percina caprodes* (Rafinesque 1818) [43,44] and the mottled sculpin *Cottus bairdii* (Girard 1850) [4,36]. The round goby has been described as out-competing native mottled sculpin for preferred habitats and disrupting its reproduction [4,36]. Rapid decline of the river bullhead *Cottus perifretum* [45] was observed by van Kessel et al. [46] following round goby colonization in the river Meuse in the Netherlands. Field and laboratory studies have revealed that cottiids, representative of small benthic fishes, might be especially vulnerable to gobiid impact [3,29]. Hence, we assume adverse effects on other cottiids similar to that on the European bullhead used in our study. These reports may not be conclusive: Janáč et al. [32], in long-term monitoring of rip-rap habitats along the middle Danube, observed that the European bullhead maintained relatively strong reproducing populations despite the long-term presence of invasive gobiids. However, information concerning the European bullhead populations prior to the gobiid invasion is not available.

Similarities between the round goby and European bullheads include size, bottom-dwelling habits, spawning grounds preferences, habitat use, feeding areas, and food type [32,47,48]. Similar to the round goby, the European bullhead is a solitary and territorial fish [49], and both species are reported to be nocturnal [50,51]. During the normal behavior period, we found both species to spend significantly less time outside shelter in daylight than in darkness, while, during the acclimation/exploration period, both were more active in light due to stress.

The European bullhead is believed to primarily inhabit small streams with a strong current [26,52] and to not actively migrate [52], while the round goby is associated with deep, lentic, slowly flowing waters and shows migratory behavior [53,54]. Some evidence suggests that the species can live in syntropy and share habitats [2,32,55,56,57]. In most cases, the round goby is a more successful species in natural conditions than the European bullhead and other cottiids [39,40]. It is generally assumed that the main drivers of the successful competition of the round goby have higher adaptability to alternative food sources as showing non-selectivity when consuming various size preys (e.g., macrozoobenthos crayfish) [58,59], fast growth and early sexual maturation, leading to rapid formation of dense populations [9,35]. Little is known about the effects of flow velocity and shelter availability on competition between the European bullhead and round goby, and these factors may be crucial for their potential co-occurrence in freshwaters and even for the persistence of the European bullhead in European waters. Our hypothesis that the round goby would exhibit a higher activity level than the European bullhead was confirmed by results showing greater time spent in movement during all investigated time periods and especially during the normal behavior period in light.

Kessel et al. [29], in separate-species experiments, reported *C. perifretum* to show strong preference for shelter, whereas the round goby displayed a more generalist pattern, exploring and moving while occupying various habitat types.

We also initially assumed that the round goby would adapt more readily during the acclimatization period. This was not confirmed: both species needed more time to display stabilized and consistent movement patterns under the novel conditions of the trial.

Although most ethological studies have been performed in still water conditions, Jermacz et al. [9] assessed the effect of flow velocity on interactions between the non-native racer goby *Babka gymnotrachelus* (Kessler 1857) and the European bullhead. The racer goby could displace the native European bullhead from a shelter in water velocities to 0.3 m s^−1^. In our study, both species coped with the higher water velocities that occurred in the middle and upstream zones, as they spent the most time in the shelter.

Reported habitat use and preferences of the European bullhead and round goby differ among sites and studies. In England, the European bullhead is reported to prefer depths of 0.10–0.30 m and velocities 0.0–0.2 m s^−1^ [57,60,61], whereas Knaepkens et al. [61] in Belgium observed a preference for depths of 0.23–0.44 m and velocities 0.0–0.6 m s^−1^. Our results also confirmed the preference of the European bullhead for more rapidly running water compared to the round goby. The European bullhead spent more time in the escape zone (water velocity ~ 0.4 m s^−1^) during normal behavior tracking in the light period than did the round goby, while the round goby spent more time in the quiet zone (~0.02 m s^−1^) during the normal behavior tracking in both dark and light compared to the European bullhead.

The hypothesis of higher food intake of the round goby is rejected, since the species ate an equal number of larvae. This finding also indicates that both species are able to cope with high water velocities, as flow velocity in the feeding zone exceeded 0.7 m s^−1^. However, less time spent in the feeding area while ingesting the same quantity of feed in the round goby implies more targeted movement toward the feeding zone.

The round goby showed more consistent behavior during the normal behavior period in light, while the European bullhead demonstrated more consistent behavior during the normal behavior period in the dark.

Comparing the behavior of the species under laboratory conditions simulating a natural habitat, the present study revealed no significant interspecific differences in behavior without competitor presence. However, some specific differences were observed, as the round goby exhibited more consistent behavior than bullhead, which tended to show more or less lively activity behavior. The slight preference for the quiet zone in the round goby and for zones of more rapid flow in the European bullhead was initially expected to occur to a greater extent than was observed.

## 5. Conclusions

Our results provided evidence that the fish need a relatively long acclimatization period for behavior stabilization. This finding should be taken into account in future ethological studies conducted under laboratory conditions. Fish behavior depends on the habitat structure and integrity, water physical and chemical properties, and the presence of other fauna, which can trigger changes in preferences and factors in adaptation to a new environment, dramatically altering direct interaction between species. The interaction of the round goby and European bullhead could possibly impact native species and increase their vulnerability to other environmental threats. In addition to suggesting future research for further evaluation of behavior and interactions of the round goby and European bullhead, we can conclude that the success of the round goby over the European bullhead will most likely not be driven by the basic behavior patterns investigated in this study. The major factors are likely to be the round goby’s higher reproduction rate and greater adaptability to often-changing environmental conditions. Our study shed light on the basic behavior of the studied species, showing very similar preferences of the native and invasive benthic fish. In case of the already widely spread invasive round goby, the implications are that European bullhead habitats are potentially susceptible to round goby invasions. Therefore, the main focus of adequate management actions should be to prevent spreading of the round goby as to ban manipulation with *N. melanostomus* and its release back into the water.

## Figures and Tables

**Table 1:** Biometric data of the round goby (RG) *Neogobius melanostomus* and European bullhead (EB) *Cottus gobio*. Total length (TL), standard length (SL), weight (W), and the average number of *Sarcophaga carinaria* larva consumed during the 28 h experiment. Data are mean ± SD. Same letter in the superscripts within the rows indicate no significant differences between species in measured parameters.

Species	n	TL (mm)	SL (mm)	W (g)	Eaten Larvae
RG	15 (12F-3M)	85.3 ± 11.5	70.7 ± 9.7	8.5 ± 5.9	4.7 ± 5.0 ^a^
EB	15	93.3 ± 13.3	78.7 ± 12.5	8.3 ± 3.9	4.7 ± 5.1 ^a^

**Table 2:** The distance moved (cm), time spent outside shelter (sec and %), time spent in motion outside the shelter (sec and %), and proportion of time spent in movement (%) during the trial observed in *Neogobius melanostomus* (RG) and *Cottus gobio* (EB). SR = stress response period, EPL = exploration period in light, EPD = exploration period in darkness; NPD = normal behavior period in darkness, NPL = normal behavior period in light. Values with different letters within rows indicate significant intraspecific differences (α = 0.05). Data are mean ± SD.

Species	Time Period	Distance Moved (cm)	Outside the Shelter (s)	Outside the Shelter (%)	Motion outside the Shelter (sec)	Motion outside the Shelter (%)	Active Movement during the Trial (%)
RG	SR	1129.5 ± 1456.1 ^a^	2671.5 ± 2693.0 ^a^	18.6 ± 18.7 ^c^	1058.0 ± 881.6 ^a^	53.6 ± 27.4 ^b^	7.3 ± 6.1 ^c^
EB	SR	1551.4 ± 1624.4 ^a^	3864.8 ± 3077.1 ^a^	26.8 ± 21.4 ^b,c^	1091.9 ± 975.5 ^a^	35.5 ± 23.4 ^b^	7.6 ± 6.8 ^c^
RG	EPL	3194.2 ± 2871.4 ^a^	7720.0 ± 5265.8 ^a^	53.6 ± 36.6 ^b^	2556.5 ± 2603.6 ^a^	35.5 ± 28.5 ^b^	17.8 ± 18.1 ^b^
EB	EPL	3773.8 ± 3342.2 ^b^	8242.5 ± 5117.0 ^a^	57.2 ± 35.5 ^c^	2415.2 ± 1716.5 ^b^	39.8 ± 28.1 ^c^	16.8 ± 11.9 ^d^
RG	EPD	2478.8 ± 2410.7 ^a^	3217.1 ± 4501.2 ^a^	22.3 ± 31.3 ^c,d^	1119.2 ± 1600.3 ^a,b^	46.7 ± 27.5 ^b,c^	7.8 ± 11.1 ^d^
EB	EPD	2951.4 ± 2579.4 ^a^	2030.4 ± 2217.1 ^b^	14.1 ± 15.4 ^d^	659.7 ± 579.3 ^b^	47.2 ± 32.1 ^c^	4.6 ± 4.0 ^d^
RG	NPD	2294.0 ± 3761.2 ^b^	10622.4 ± 8817.1 ^a^	36.8 ± 30.7 ^c^	4234.0 ± 5488.9 ^b^	44.5 ± 27.1 ^c^	14.6 ± 18.9 ^d^
EB	NPD	1207.1 ± 1234.3 ^c^	10411.7 ± 10275.1 ^a^	36.1 ± 35.6 ^d^	3640.2 ± 4753.0 ^b^	45.4 ± 25.1 ^d^	12.6 ± 16.5 ^e^
RG	NPL	1772.6 ± 2797.5 ^b^	9796.1 ± 8455.6 ^a^	34.1 ± 29.4 ^c,d^	4933.0 ± 4637.1 ^a^	52.5 ± 26.1 ^c^	17.1 ± 16.2 ^d^
EB	NPL	1828.3 ± 2117.4 ^b^	6828.0 ± 7682.5 ^a^	23.7 ± 26.7 ^d^	2864.3 ± 3243.2 ^a,b^	54.8 ± 22.9 ^c^	9.9 ± 11.3 ^d^

## Data Availability

The data presented in this study are available on request from the corresponding author.

## Supplementary Materials

The following are available online at https://www.mdpi.com/article/10.3390/biology10090821/s1, Table S1: The mean values measured at 20 water velocity points (WV in m sec^−1^ measurement points MP (A1-H1) in all three (1–3) experimental channels (EC). Data are mean ± SD., Figure S1: Simple linear relationship between number of fly larvae consumed and total time spent in feeding zone by round goby and European bullhead using the default *lm* function in the R statistical program (R Core Team, 2020).
