# Supplementary material for: Comparison of Behavior and Space Use of the European Bullhead Cottus gobio and the Round Goby Neogobius melanostomus in a Simulated Natural Habitat
Source: Biology (Basel). 2021 Aug 24;10(9):821. doi: 10.3390/biology10090821 (PMC8471795; doi:10.3390/biology10090821)
Supplement: Supplementary file 1 [file biology-10-00821-s001.zip › Supplement 1.pdf]

Supplementary material 1.

**Table S1.** The mean values measured at 20 water velocity points (WV in m $sec^{-1}$ measurement points MP (A1-H1) in all three (1-3) experimental channels (EC). Data are mean ± SD.

| EC | MP | WV | MP | WV | MP | WV | MP | WV | MP | WV | MP | WV | MP | WV | MP | WV |
|---|---|---|---|---|---|---|---|---|---|---|---|---|---|---|---|---|
| 1 | A1 | 0.78±0.05 | B1 | 0.20±0.08 | C1 | 0.23±0.06 | D1 | 0.53±0.04 | E1 | 0.56±0.04 | F1 | 0.03±0.03 | G1 | 0.11±0.04 | H1 | 0.21±0.04 |
| | A2 | 0.10±0.03 | B2 | 0.32±0.05 | C2 | 0.31±0.06 | | | | | F2 | 0.33±0.10 | G2 | 0.32±0.03 | H2 | 0.36±0.04 |
| | A3 | 0.82±0.06 | B3 | 0.34±0.06 | C3 | 0.30±0.04 | | | | | F3 | 0.56±0.03 | G3 | 0.46±0.04 | H3 | 0.35±0.06 |
| 2 | A1 | 0.79±0.04 | B1 | 0.34±0.04 | C1 | 0.28±0.03 | D1 | 0.55±0.04 | E1 | 0.59±0.02 | F1 | 0.02±0.02 | G1 | 0.12±0.04 | H1 | 0.18±0.04 |
| | A2 | 0.15±0.05 | B2 | 0.25±0.04 | C2 | 0.31±0.02 | | | | | F2 | 0.42±0.11 | G2 | 0.36±0.07 | H2 | 0.33±0.04 |
| | A3 | 0.89±0.06 | B3 | 0.40±0.08 | C3 | 0.30±0.05 | | | | | F3 | 0.56±0.04 | G3 | 0.40±0.02 | H3 | 0.31±0.03 |
| 3 | A1 | 0.81±0.05 | B1 | 0.26±0.04 | C1 | 0.26±0.03 | D1 | 0.52±0.04 | E1 | 0.58±0.02 | F1 | 0.01±0.01 | G1 | 0.14±0.04 | H1 | 0.20±0.04 |
| | A2 | 0.08±0.03 | B2 | 0.32±0.03 | C2 | 0.33±0.04 | | | | | F2 | 0.41±0.11 | G2 | 0.39±0.05 | H2 | 0.35±0.03 |
| | A3 | 0.86±0.05 | B3 | 0.35±0.04 | C3 | 0.29±0.06 | | | | | F3 | 0.58±0.02 | G3 | 0.43±0.08 | H3 | 0.33±0.04 |
