# Supplementary material for: Comparison of Behavior and Space Use of the European Bullhead Cottus gobio and the Round Goby Neogobius melanostomus in a Simulated Natural Habitat
Source: Biology (Basel). 2021 Aug 24;10(9):821. doi: 10.3390/biology10090821 (PMC8471795; doi:10.3390/biology10090821)
Supplement: Supplementary file 1 [file biology-10-00821-s001.zip › Supplement 2-novi.pdf]

Supplementary data 2.

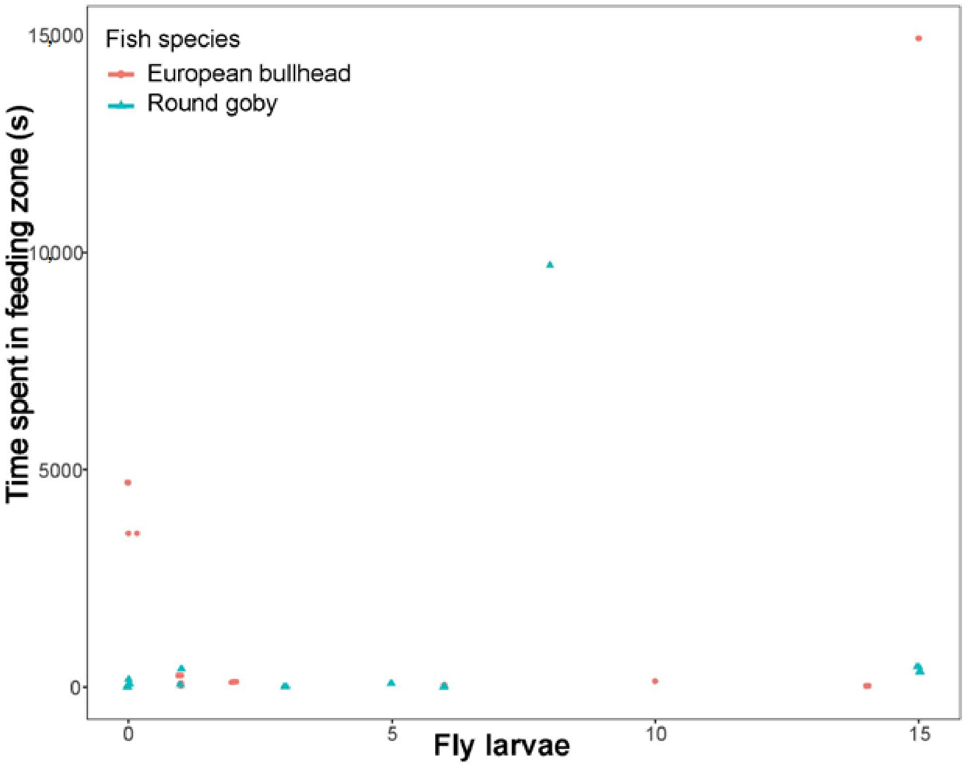

Figure S1. Simple linear relationship between number of fly larvae consumed and total time spent in feeding zone by round goby and European bullhead using the default *lm* function in the R statistical program (R Core Team, 2020).
